# Supplementary material for: Aborting meiosis allows recombination in sterile diploid yeast hybrids
Source: Nat Commun. 2021 Nov 12;12:6564. doi: 10.1038/s41467-021-26883-8 (PMC8589840; doi:10.1038/s41467-021-26883-8)
Supplement: Supplementary file 1 — Supplementary Information [file 41467_2021_26883_MOESM1_ESM.pdf]

Supplementary information files

Aborting meiosis allows recombination in sterile diploid yeast hybrids

Simone Mozzachiodi[1,2,†], Lorenzo Tattini[1,†], Agnes Llored[1], Agurtzane Irizar[1], Neža Škofljanc[1], Melania D'Angiolo[1], Matteo De Chiara[1], Benjamin P. Barré[1], Jia-Xing Yue[1,#], Angela Lutazi[1], Sophie Loeillet[3], Raphaelle Laureau[3,$], Souhir Marsit[3,4,^], Simon Stenberg[5], Benoit Albaud[6], Karl Persson[5], Jean-Luc Legras[4], Sylvie Dequin[4], Jonas Warringer[5], Alain Nicolas[1,2,3] and Gianni Liti[1,*]

Affiliations

[1]Université Côte d'Azur, CNRS, INSERM, IRCAN, Nice, France. [2]Meiogenix, 38, rue Servan, Paris 75011, France. [3]Institut Curie, Centre de Recherche, CNRS-UMR3244, PSL Research University, Paris 75005, France. [4]SPO, Université Montpellier, INRAE, Montpellier SupAgro, Montpellier, France. [5]Department of Chemistry and Molecular Biology, University of Gothenburg, Gothenburg, Sweden. [6]Institut Curie, ICGEX NGS Platform, Paris 75005, France.

Notes

[†]These authors contributed equally to this work. [#]Current address: State Key Laboratory of Oncology in South China, Collaborative Innovation Center for Cancer Medicine, Sun Yat-sen University Cancer Center, Guangzhou, China. [$]Current address: Department of Genetics and Development, Hammer Health Sciences Center, Columbia University Medical Center, New York, NY, USA. [^]Current address: Département de Biologie Chimie et Géographie, Université du Québec à Rimouski, Rimouski, Québec, Canada. [*]Correspondence and requests for materials should be addressed to G.L. (gianni.liti@unice.fr)

The supplementary information file includes:

- Supplementary notes
- Supplementary figures 1-9

Supplementary notes

Testing for “recombination rates”-“meiotic progression” and “recombination rates”-“sequence divergence” correlations

We used a simple linear model to fit the recombination rates data (measured as both LOH difference and LOH ratio) with the genome-wide sequence divergence but we did not find significant evidence to support the regression (“LOH ratio”-“sequence divergence” correlation: R=-0.36, p=0.23, “LOH difference”-“sequence divergence” correlation: R=-0.5, p=0.08; R is the Pearson correlation coefficient). We tested the correlation between the LOH ratio and the meiotic progression (“MI+MII cells”) as well as the correlation between the LOH difference and the meiotic progression. Both tests showed strong correlation, as reported in **Fig. 2e**.

We also used a multiple linear model taking into account two explanatory variables, i.e. the meiotic progression (“MI+MII cells”) and the sequence divergence (“SequenceDivergence”), and the LOH ratio as the explained variable. The sequence divergence did not show statistical evidence to support the regression. Thus, the multiple linear model could be reduced to the simple linear model that we used in **Fig. 2e**.

The values reported in the following table are: the estimated coefficient (“Estimate”), its standard error (“Std. Error”), the t-statistic (“t value”) and the corresponding two-sided *p*-value (“Pr(>|t|)”).

#Multiple linear regression using the relative LOH ratio

Coefficients:

	Estimate	Std. Error	t value	Pr(>|t|)
(Intercept)	5.246e-02	2.909e-01	0.180	0.860
MI+MII cells	6.445e-02	9.019e-03	7.146	3.12e-05 ***
SequenceDivergence	-3.983e-07	2.876e-07	-1.385	0.196

Signif. codes: 0 ‘***’ 0.001 ‘**’ 0.01 ‘*’ 0.05 ‘.’ 0.1 ‘ ’ 1

Residual standard error: 0.5225 on 10 degrees of freedom

Multiple R-squared: 0.8504, Adjusted R-squared: 0.8204

F-statistic: 28.42 on 2 and 10 DF, *p*-value: 7.501e-05

Finally, sequence divergence was found a marginally significant explanatory variable for the LOH difference while the meiotic progression was confirmed as the major explanatory variable.

#Multiple linear regression using the LOH difference

Coefficients:

	Estimate	Std. Error	t value	Pr(>|t|)
(Intercept)	1.419e-02	3.618e-02	0.392	0.703148
MI+MII cells	5.152e-03	1.122e-03	4.593	0.000991 ***
SequenceDivergence	-9.19e-08	3.578e-08	-2.568	0.027967 *

Signif. codes: 0 ‘***’ 0.001 ‘**’ 0.01 ‘*’ 0.05 ‘.’ 0.1 ‘ ’ 1

Residual standard error: 0.065 on 10 degrees of freedom

Multiple R-squared: 0.7595,   Adjusted R-squared: 0.7114

F-statistic: 15.79 on 2 and 10 DF, p-value: 0.0008047

LOH occurrence in collinear and non-collinear chromosomes

For each chromosome (excluding chromosome II), we calculated the mean number of events per sample per Mbp detected in the ScMA/ScNA hybrids (**Supplementary Table 15**) and performed the Welch’s t-test (two-sided) to test if the mean number of events detected (0.664 ± 0.198 and 0.525 ± 0.101, respectively) was different for the collinear chromosomes with respect to the non-collinear ones (i.e. chromosomes VII, VIII, X, XI and XIII). We obtained p-value = 0.095 thus rejecting the alternative hypothesis and proving that the collinear and the non-collinear chromosomes in the ScMA/ScNA RTGs recombined with a similar frequency.

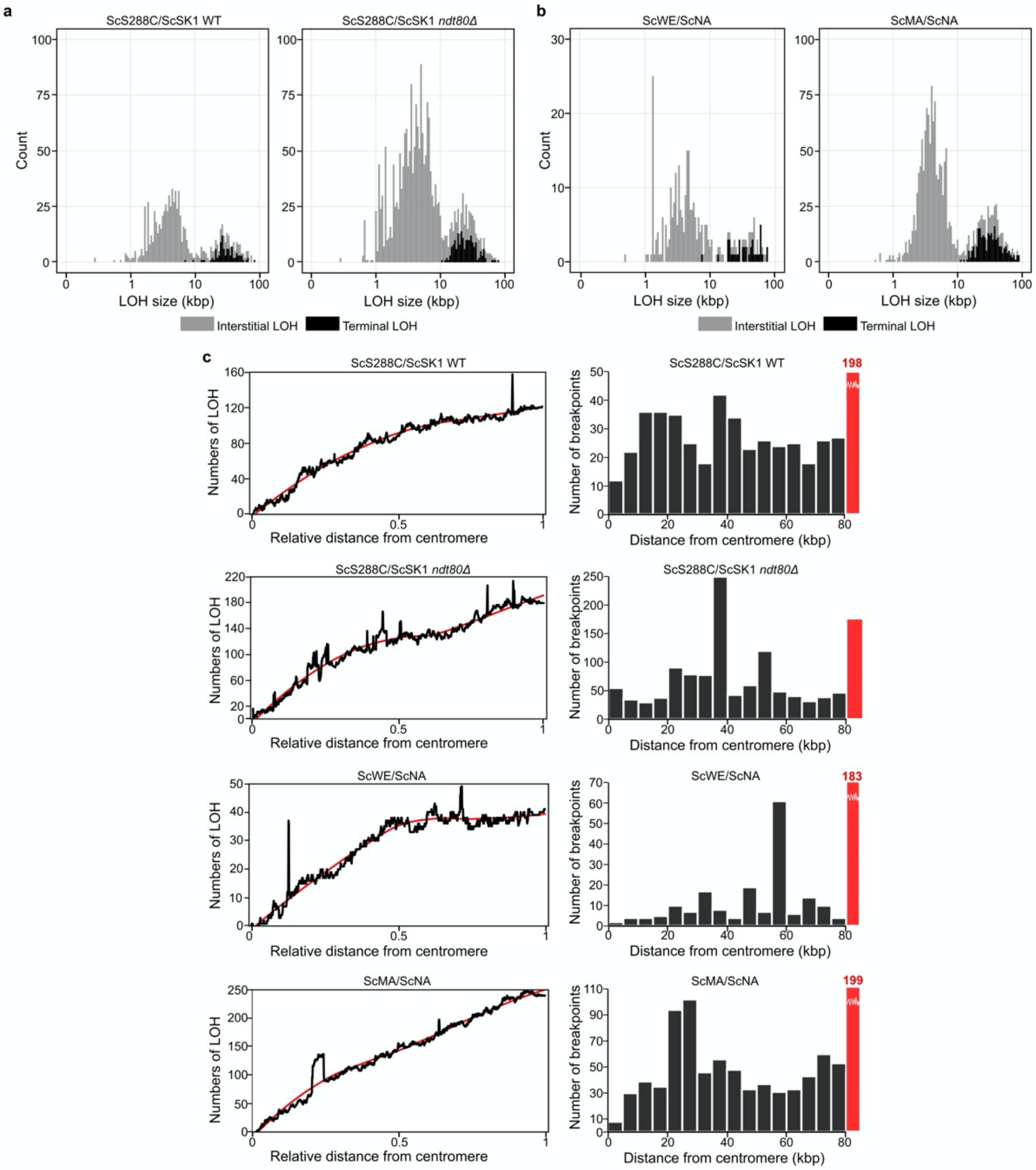

Supplementary Figure 1. LOH size and chromosomal position. (**a**) Size distribution of interstitial (grey) and terminal (black) LOH events for the ScS288C/ScSK1 WT and *ndt80*Δ RTGs. The x-axis is in logarithmic scale. (**b**) Size distribution of interstitial (grey) and terminal (black) LOH events for the intraspecies (light brown) and interspecies (dark brown) RTGs. The x-axis is in logarithmic scale. (**c**) Left panels: cumulative number of LOHs versus the relative distance from the centromere. For each background, the number of LOH events spanning 1000 bins of width 0.001 is reported as a black line. The plots represent an aggregate analysis for all chromosomes except for events lying on chromosome II that were filtered out since positions of LOH in this chromosome were constrained by the presence of the *LYS2/URA3* system (see text). The smoothing of the data (red line) serves as an eye guide. Right panels: bar plots of the number of breakpoints in a 80 kbp window (5 kbp bin size) around all the centromeres (excluding chromosome II) for the intraspecies RTGs. Red bars represent the whole-genome median. The values exceeding the y-axis upper limit are annotated on top of the corresponding bar.

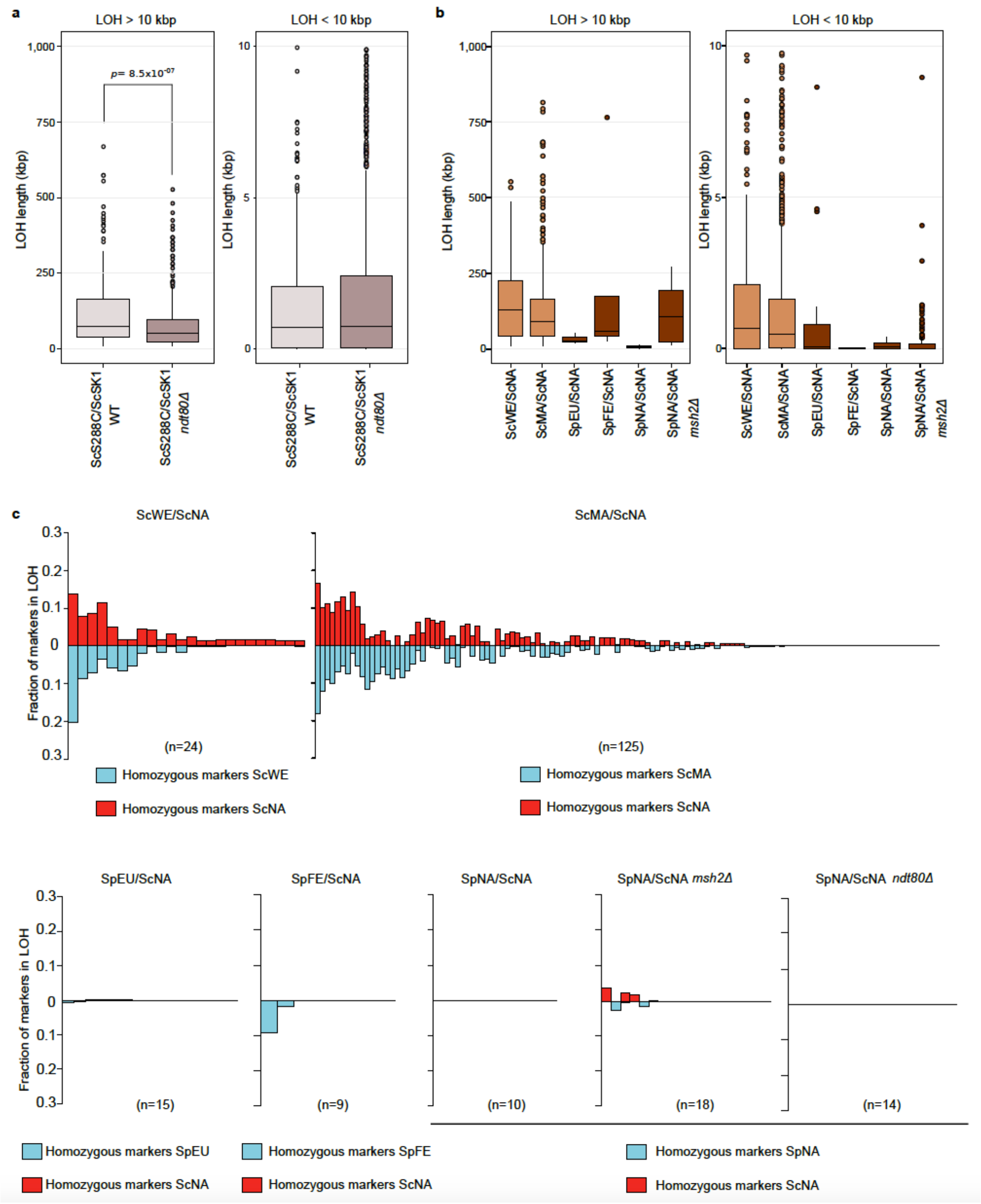

Supplementary Figure 2. Overview of LOH length and heterozygous/homozygous markers in RTG clones. (**a**) Boxplots of ScS288C/ScSK1 (*n*=219 left, *n*=603 right) and S288C/SK1 *ndt80Δ* (*n*=415 left, *n*=1699 right) LOHs (left > 10 kbp and right < 10 kbp). The *p*-value refers to a two-tailed Wilcoxon rank-sum test. The same test performed only on the mother cells of the *ndt80Δ* and WT populations confirmed this result (one sided Wilcoxon rank-sum test, *p*-value=2.34×10^{-8}) (**b**) Boxplots of the LOH (left > 10 kbp and right < 10 kbp) for the intraspecies (ScWE/ScNA n=72, ScMA/ScNA n=417, left; ScWE/ScNA n=231 ScMA/ScNA n=1172, right) and interspecies hybrids (SpEU/ScNA n=3, SpFE/ScNA n=5, SpNA/ScNA n=1, SpNA/ScNA *msh2Δ* n=11, left, SpEU/ScNA n=31, SpFE/ScNA n=6, SpNA/ScNA n=8, SpNA/ScNA *msh2Δ* n=229, right) coloured according to **Fig. 2**. (**c**) RTG clones from intraspecies hybrids display high heterogeneity in the genome fraction in LOH (upper panels). No LOH bias towards one of the two hybrid's subgenomes for intraspecies hybrids was observed (Welch's t-test two-sided ScWE/ScNA, *p*-value = 0.8, Welch's t-test two-sided ScMA/ScNA, *p*-value = 0.74). Interspecies hybrids show a lower magnitude of genome in LOH (lower panels). The boxplot is defined as follows: the box is delimited by the first quartile (Q1) and the third quartile (Q3). The line that separates the box is the median. Whiskers are defined as: upper whisker = min(max(x), Q3 + 1.5 * IQR); lower whisker = max(min(x), Q1 – 1.5 * IQR), where: x is the data, Q1 is the first quartile, Q3 is the third quartile, and IQR is the inter-quartile range (IQR = Q3 - Q1).

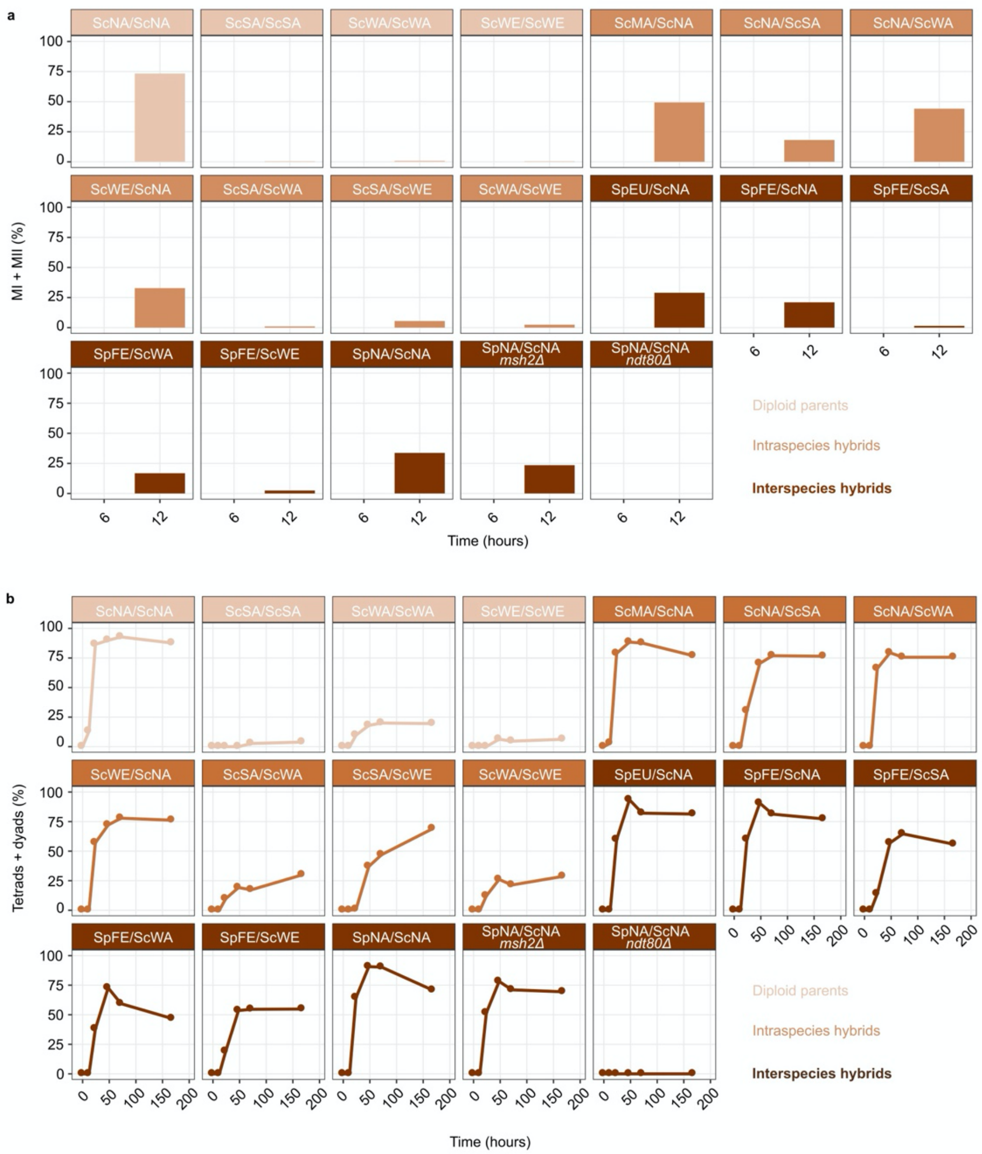

Supplementary Figure 3. Sporulation dynamics across the strain panel. (**a**) Meiotic progression measured by DAPI staining as the combined percentage of cells with 2 nuclei (MI) and 4 nuclei (MII). None of the samples had 2 or 4 nuclei at T0 (not reported) and T6. (**b**) Sporulation efficiency measured as the combined percentage of tetrads and dyads observed on the total number of counted cells.

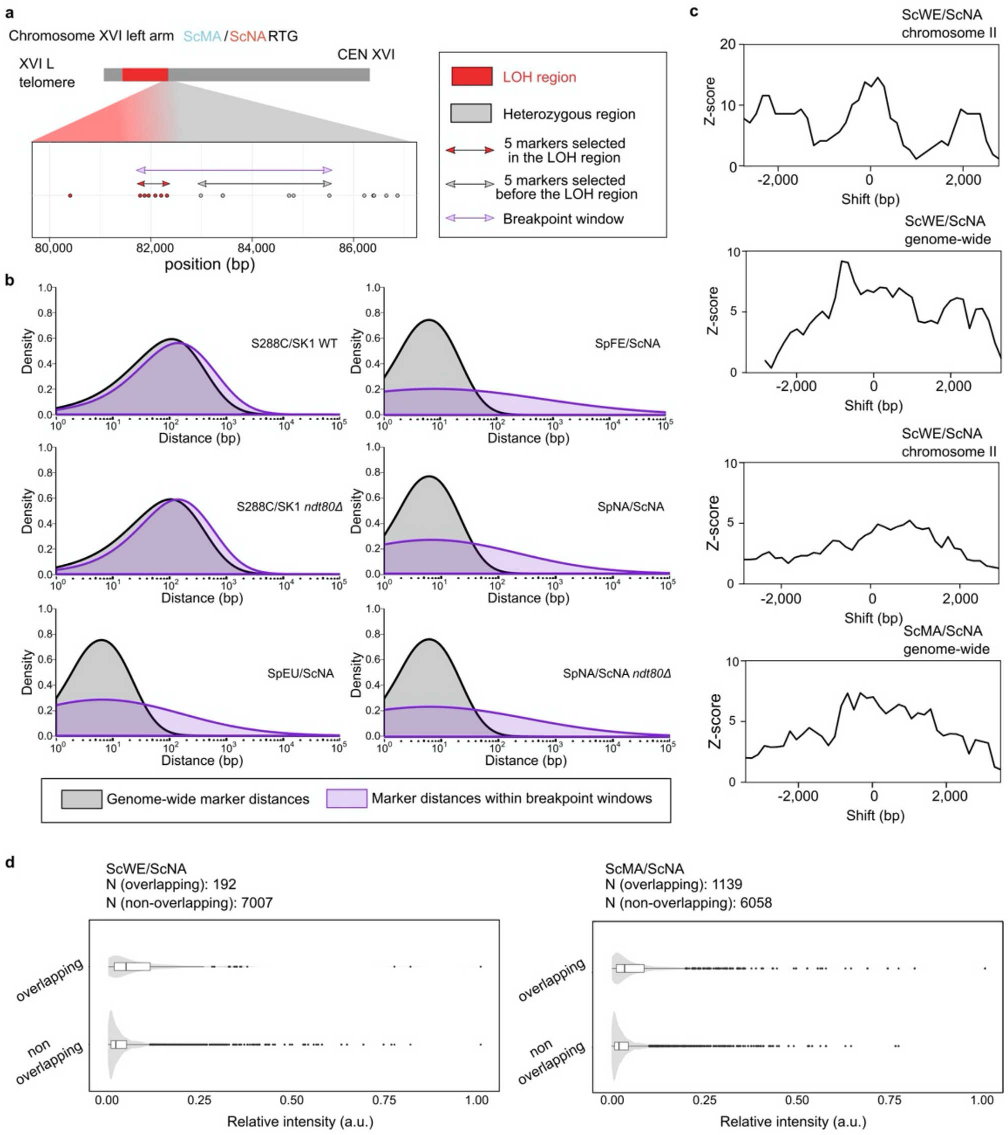

Supplementary Figure 4. LOH breakpoints are enriched in low-heterozygosity regions and nearby meiotic recombination hotspots. (a) Zoom-in of an LOH breakpoint window showing which markers were used to evaluate the local heterozygosity (see **Fig. 3** and **Fig. S4b**). LOH breakpoint windows comprise the 5 heterozygous markers and the 5 homozygous markers closer to the breakpoint. **(b)** Distribution of marker distances genome-wide (grey) and in LOH breakpoint windows (purple) across different hybrids. LOH breakpoint regions are defined e.g. as the genomic interval between the first homozygous marker of an LOH region and the closest flanking marker which does not belong to the same LOH region. **(c)** Z-score of the statistical test for LOH breakpoints and recombination hotspots association on chromosome II and genome-wide for the ScWE/ScNA and ScMA/ScNA RTG-evolved samples. The Z-score is plotted as a function of the shift (in bp) of the recombination hotspots regions. **(d)** Distributions of Spo11 oligos relative intensity in regions overlapping the LOH breakpoints against the intensity in regions that do not overlap the LOH breakpoints for the ScWE/ScNA and the ScMA/ScNA crosses. The boxplot is defined as follows: the box is delimited by the first quartile (Q1) and the third quartile (Q3). The line that separates the box is the median. Whiskers are defined as: upper whisker = min(max(x), Q3 + 1.5 * IQR); lower whisker = max(min(x), Q1 – 1.5 * IQR), where: x is the data, Q1 is the first quartile, Q3 is the third quartile, and IQR is the inter-quartile range (IQR = Q3 - Q1).

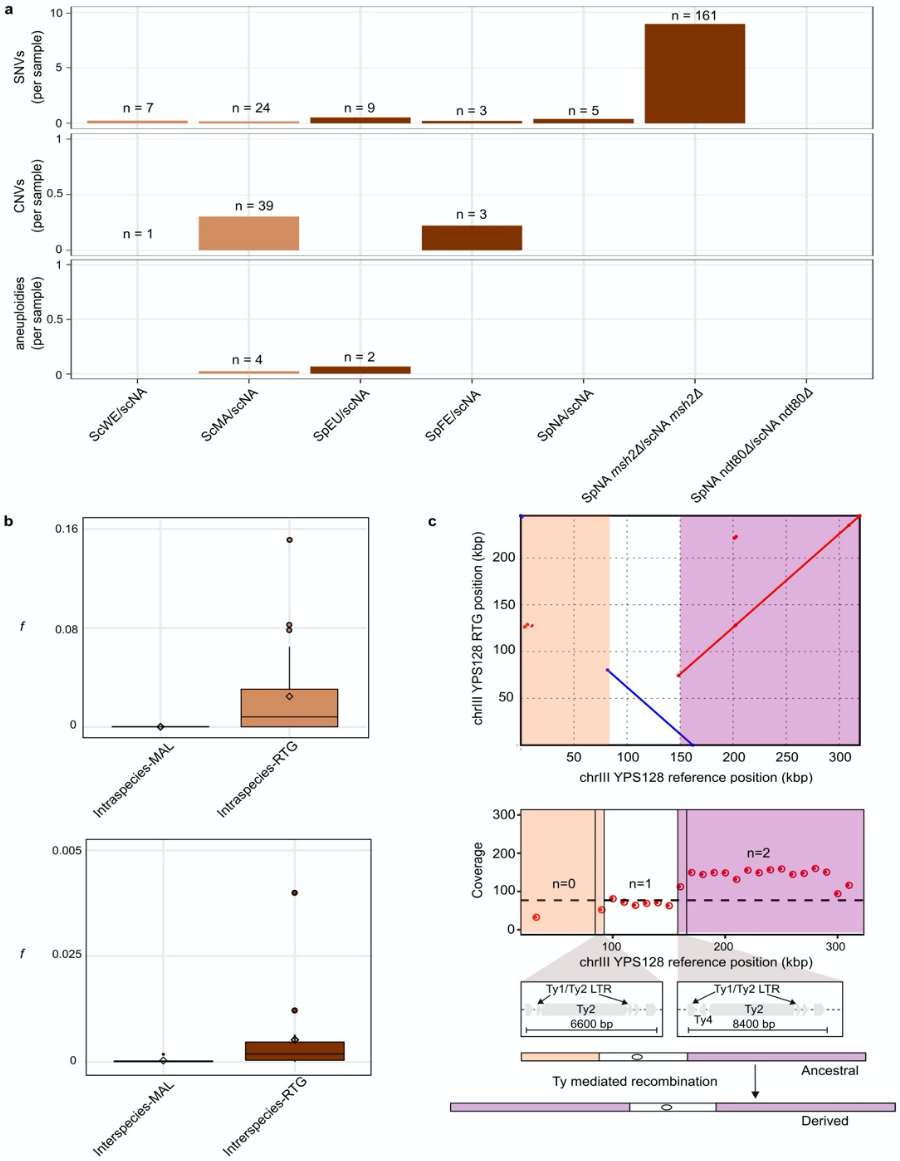

Supplementary Figure 5. Mutational landscape upon RTG. (**a**) Bar plots report the number of single nucleotide variants (SNVs), copy number variations (CNVs) and aneuploidies per sample; n is the total number of events. Genomes of evolved RTG clones are remarkably stable with no significant increase of aneuploidies, single nucleotide variants (SNVs) and copy number variations (CNVs). The increased SNVs in the SpNA/ScNA *msh2Δ* hybrid is expected given its mutator phenotype. Massive CNVs in the ScMA/ScNA samples arise as a result of recombination between non-collinear chromosome arms. (**b**) Top panel: LOH genomic impact on intraspecies ScWE/ScNA hybrids evolved through mutation accumulation lines (MAL) (*n*=8) and RTGs (*n*=24); *f* is the fraction of genome in LOH per genome per bottleneck. Events lying on chromosome II of RTG samples were filtered out. Mean values are reported as diamonds. Bottom panel: LOH genomic impact for interspecies SpEU/ScWE hybrids evolved through MAL (*n*=8) and the SpEU/ScNA RTGs (*n*=10). Mean values are reported as diamonds. (**c**) Top panel: dot plot of chromosome III from Nanopore long-read sequencing and *de novo* assembly of an RTG clone which underwent a complex rearrangement resulting in loss of the distal part of the left arm, inversion of the region encompassing the centromere and duplication of part of the right arm. The Illumina short-read coverage analysis supports this rearranged structure, which was likely due to a Ty-mediated mechanism (bottom panel; n is number of copies). The boxplot is defined as follows: the box is delimited by the first quartile (Q1) and the third quartile (Q3). The line that separates the box is the median. Whiskers are defined as: upper whisker = min(max(x), Q3 + 1.5 * IQR); lower whisker = max(min(x), Q1 – 1.5 * IQR), where: x is the data, Q1 is the first quartile, Q3 is the third quartile, and IQR is the inter-quartile range (IQR = Q3 - Q1).

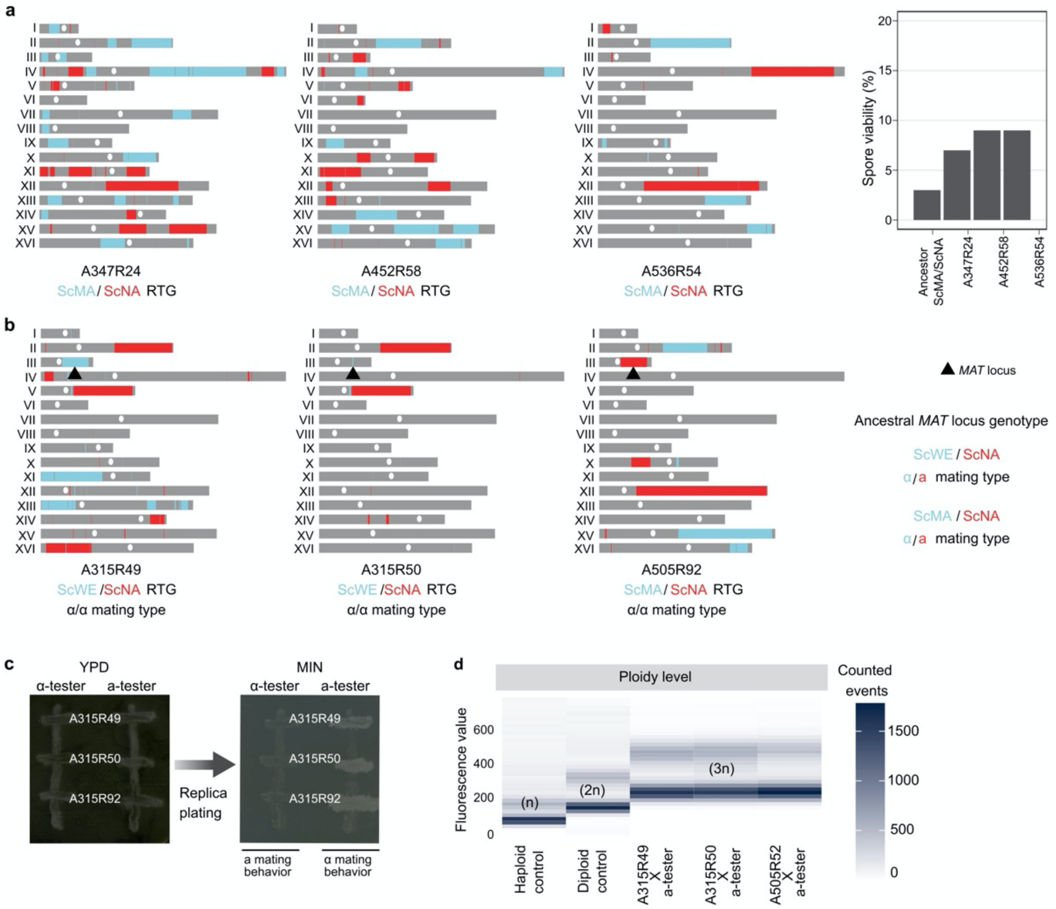

Supplementary Figure 6. RTG partially rescues hybrid fertility and can lead to polyploidization. (**a**) LOH landscapes of 3 selected ScMA/ScNA RTGs clones (left) and their respective spore viability compared to the ancestral hybrid (bar plot, right). (**b**) LOH landscape of two ScWE/ScNA RTGs and one ScMA/ScNA RTG with α/α mating type. (**c**) The mating-type test shows that RTG clones with recombination encompassing the *MAT* locus have haploid-like mating behaviour and can generate polyploid strains if mated with a partner of the opposite mating-type. (**d**) The genome content of the mated strains has been validated by flow-cytometry.

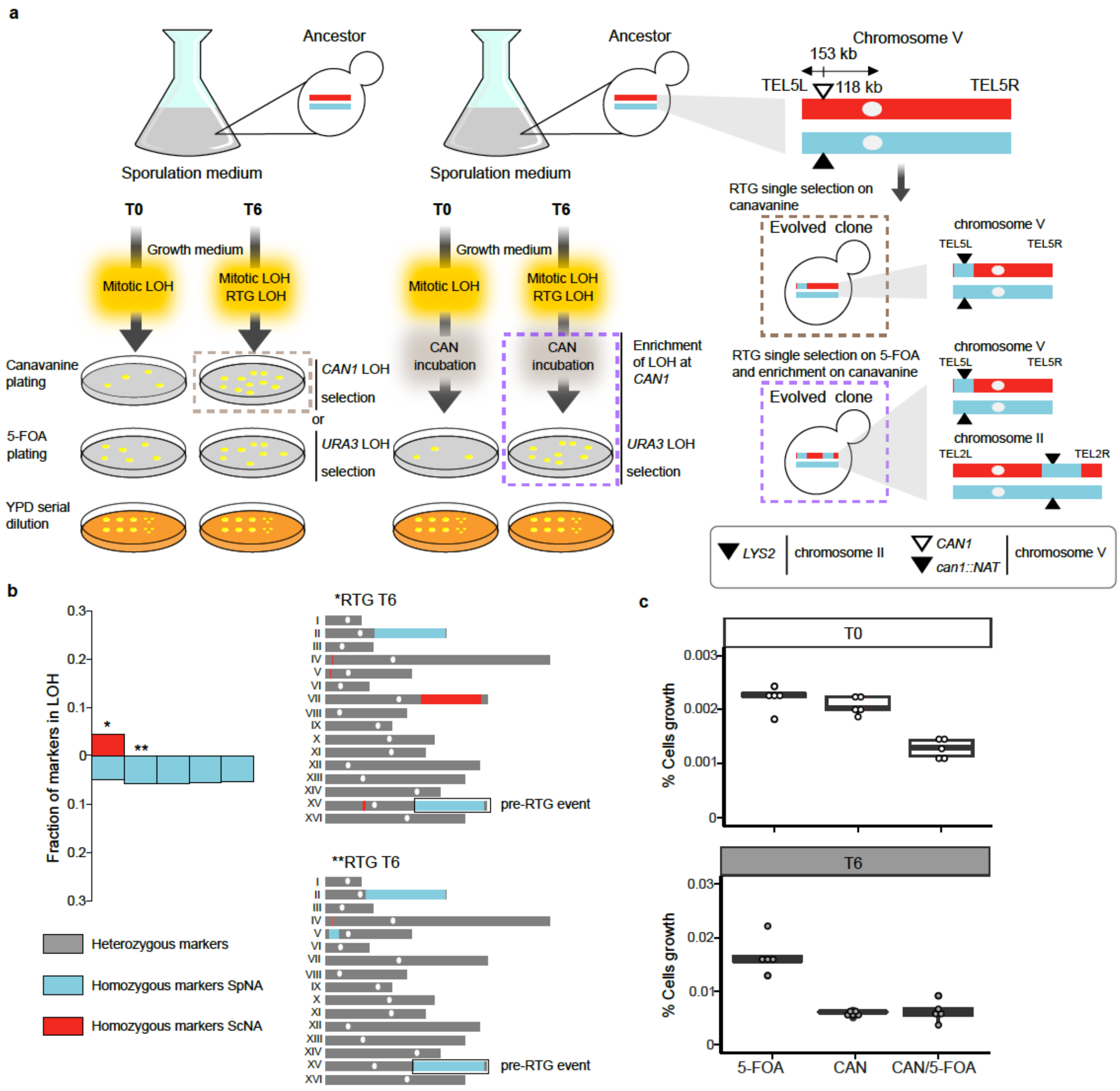

Supplementary Figure 7. Overview of the double LOH selection approach. (**a**) Left panel: RTG protocol used for the single selection approach performed either with 5-FOA or canavanine. Central panel: RTG protocol used for the enrichment of a second recombination event when cells are grown on canavanine before plating on 5-FOA. Right panel: sketch of the LOH recombination selected when plating on canavanine (brown box) and the event enriched upon canavanine incubation and plating on 5-FOA (purple box). The *CAN1* gene is deleted in the *S. paradoxus* subgenome (light blue) so that the two selectable markers promote LOH in the same direction to avoid any genetic incompatibility effect by selecting two LOHs toward different subgenomes. (**b**) Fraction of markers in LOH in the sequenced RTG isolated with the double selection approach and LOH map of two RTGs sequenced, the asterisks match the respective LOH map on the right panel. (**c**) Percentage of cells growing on 5-FOA or canavanine plates upon RTG. Three experiments are reported each performed in n=5 biological replicates: single selection approach (5-FOA and canavanine) and double selection approach. T0 = no sporulation induction, T6 = 6 hours of sporulation induction. The incubation in canavanine does not kill the cells but it allows the growth of only those having resistance to the drug thus enriching cells harbouring LOH at *CAN1*. Once the cells are plated if the two events were completely independent the ratio of T6 compared to the T0 would have been expected to be the same and cells with only the LOH on chromosome II deleting *URA3* were expected to be retrieved. The boxplot is defined as follows: the box is delimited by the first quartile (Q1) and the third quartile (Q3). The line that separates the box is the median. Whiskers are defined as: upper whisker = min(max(x), Q3 + 1.5 * IQR); lower whisker = max(min(x), Q1 – 1.5 * IQR), where: x is the data, Q1 is the first quartile, Q3 is the third quartile, and IQR is the inter-quartile range (IQR = Q3 - Q1).

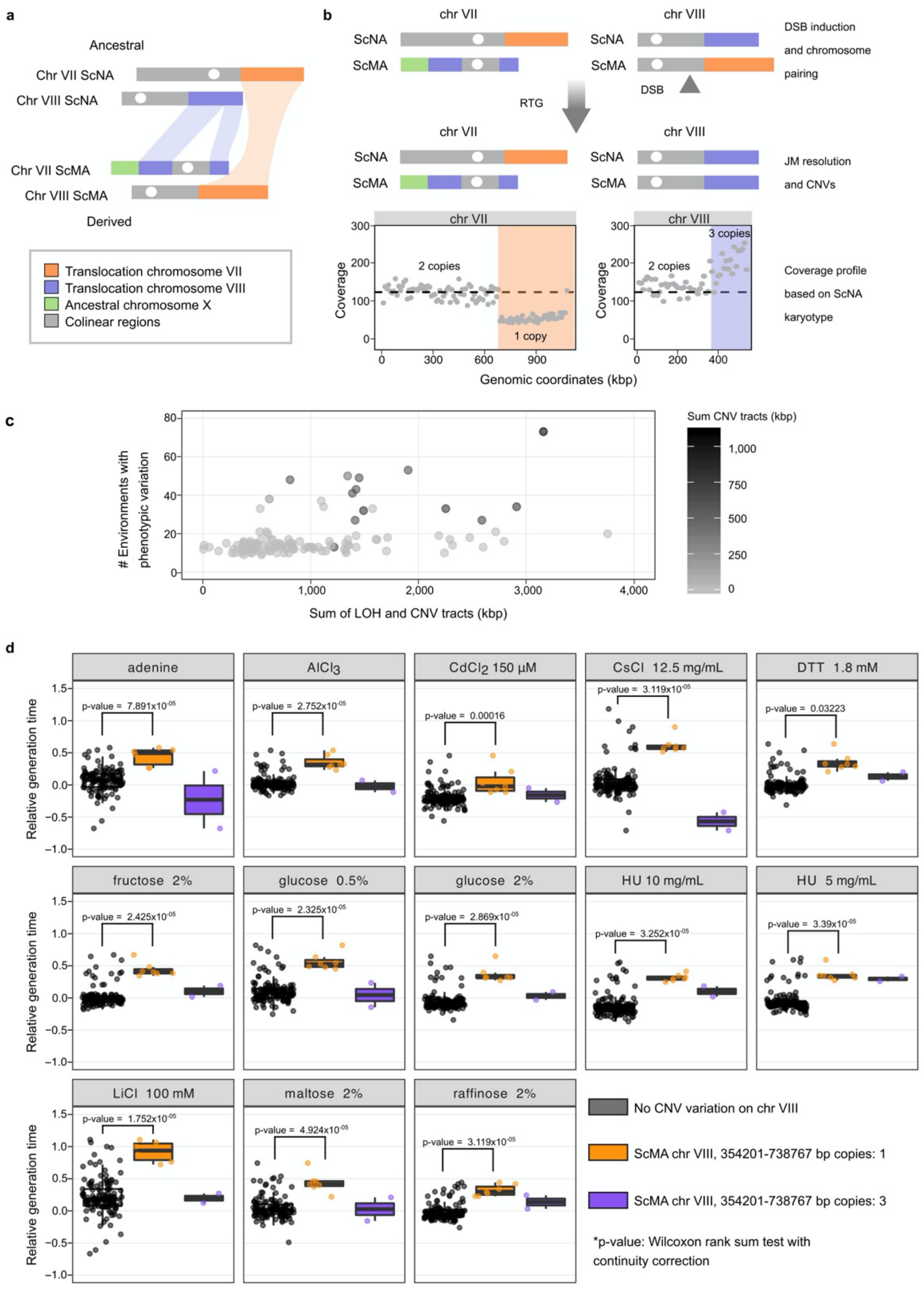

Supplementary Figure 8. Massive CNVs shape RTGs fitness. (**a**) Sketch of the non-colinear regions on chromosomes VII and VIII. The term "ancestral" refers to the ScNA karyotype whereas the term "derived" refers to the rearrangements in the ScMA karyotype. (**b**) Massive CNVs result from recombination of non-collinear chromosome arms of the subgenomes of a ScMA/ScNA hybrid. (**c**) Relationship between phenotypic variation and length of LOH and CNV tracts in the RTG samples; 2 samples were masked due to non-reliable CNV calls. (**d**) Boxplots of the relative generation time of RTGs with no CNV on chromosome VIII (black) (n=119), RTG that have lost one copy of the arm of chromosome VIII of the ScMA subgenome as a result of recombination (orange) (n=7), and RTG that have gained one copy of the arm of chromosome VIII of the ScMA subgenome as a result of recombination (purple) (n=2). The p-value represents the statistical significance calculated with a Wilcoxon rank-sum test one-tailed, with continuity correction. The boxplot is defined as follows: the box is delimited by the first quartile (Q1) and the third quartile (Q3). The line that separates the box is the median. Whiskers are defined as: upper whisker = min(max(x), Q3 + 1.5 * IQR); lower whisker = max(min(x), Q1 – 1.5 * IQR), where: x is the data, Q1 is the first quartile, Q3 is the third quartile, and IQR is the inter-quartile range (IQR = Q3 - Q1).

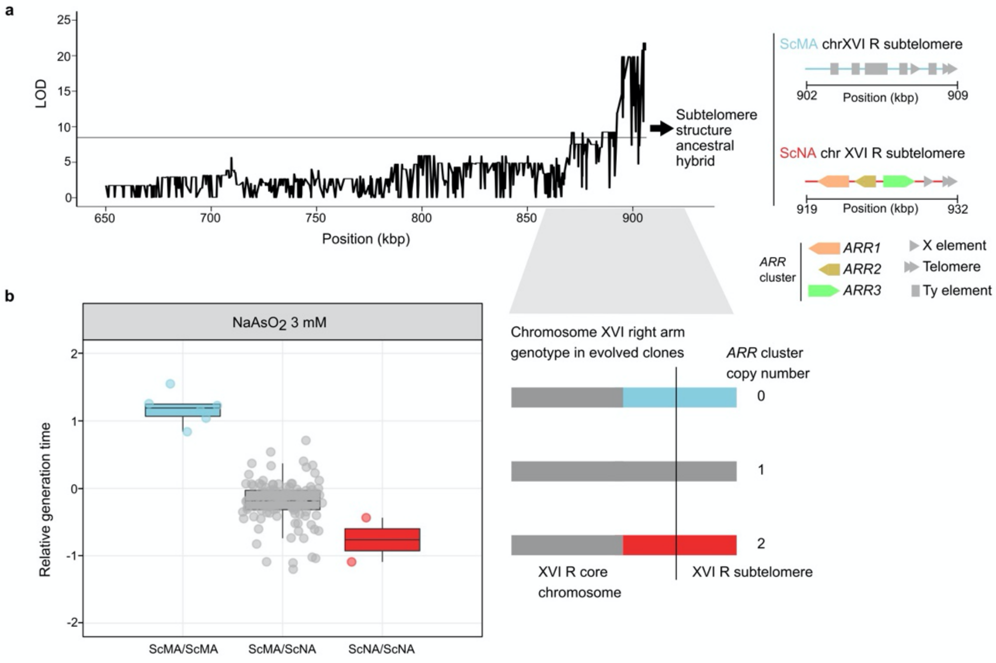

Supplementary Figure 9. Linkage analysis of arsenite resistance. (**a**) LOD score along the right arm of chromosome XVI. The horizontal line indicates the statistical significance threshold. The right sub-panel shows a zoom-in on the subtelomere structure of the two parental isolates used in the linkage mapping. The ScMA background lacks the arsenite resistance cluster *ARR1-3*. (**b**) Phenotypic variation (relative generation time) in arsenite across the ScMA/ScNA RTG samples. The data points are grouped based on the genotype of the last marker before the right subtelomere of chromosome XVI (ScMA/ScMA n=8, ScMA/ScNA n=118, ScNA/ScNA n=2). The right panel shows the different number of *ARR* clusters according to the genotype of RTG clones. The boxplot is defined as follows: the box is delimited by the first quartile (Q1) and the third quartile (Q3). The line that separates the box is the median. Whiskers are defined as: upper whisker = min(max(x), Q3 + 1.5 * IQR); lower whisker = max(min(x), Q1 – 1.5 * IQR), where: x is the data, Q1 is the first quartile, Q3 is the third quartile, and IQR is the inter-quartile range (IQR = Q3 - Q1).
