# Supplementary material for: Aborting meiosis allows recombination in sterile diploid yeast hybrids
Source: Nat Commun. 2021 Nov 12;12:6564. doi: 10.1038/s41467-021-26883-8 (PMC8589840; doi:10.1038/s41467-021-26883-8)
Supplement: Supplementary file 3 — Description of Additional Supplementary Files [file 41467_2021_26883_MOESM3_ESM.pdf]

Description of Additional Supplementary Files

File Name: Supplementary Data 1
Description: Strains used in the study

File Name: Supplementary Data 2
Description: Sporulation efficiency of the strains used in the 5-FOA assay

File Name: Supplementary Data 3
Description: Spore viability of the diploids hybrids and derived RTG samples

File Name: Supplementary Data 4
Description: Average growth percentage on 5-FOA and Canavanine for the diploid hybrids used in this work

File Name: Supplementary Data 5
Description: Environments used for phenotyping

File Name: Supplementary Data 6
Description: Summary statistics of LOHs

File Name: Supplementary Data 7
Description: Breakpoints heterozygosity statistics

File Name: Supplementary Data 8
Description: Mutfunc analysis of aminoacid mutations in the ScMA CDS

File Name: Supplementary Data 9
Description: List of samples sequenced

File Name: Supplementary Data 10
Description: LOH breakpoints association with meiotic hotspot on chrII-R

File Name: Supplementary Data 11
Description: Genome-wide LOH breakpoints association with meiotic hotspots

File Name: Supplementary Data 12
Description: Primers used in the study

File Name: Supplementary Data 13
Description: RTG analysis of reciprocal non-reciprocal markers in LOH

File Name: Supplementary Data 14
Description: Environments with BPH and WPH

File Name: Supplementary Data 15
Description: Mean number of LOHs (per sample per Mbp) detected in ScMA/ScNA hybrids
