# Supplementary material for: Aborting meiosis allows recombination in sterile diploid yeast hybrids
Source: Nat Commun. 2021 Nov 12;12:6564. doi: 10.1038/s41467-021-26883-8 (PMC8589840; doi:10.1038/s41467-021-26883-8)
Supplement: Supplementary file 19 — Reporting Summary [file 41467_2021_26883_MOESM19_ESM.pdf]

nature research

Corresponding author(s): Gianni Liti

Last updated by author(s): 2021/09/20

Reporting Summary

Nature Research wishes to improve the reproducibility of the work that we publish. This form provides structure for consistency and transparency in reporting. For further information on Nature Research policies, see our Editorial Policies and the Editorial Policy Checklist.

Statistics

For all statistical analyses, confirm that the following items are present in the figure legend, table legend, main text, or Methods section.

n/a	Confirmed	
☐	☒	The exact sample size (*n*) for each experimental group/condition, given as a discrete number and unit of measurement
☐	☒	A statement on whether measurements were taken from distinct samples or whether the same sample was measured repeatedly
☐	☒	The statistical test(s) used AND whether they are one- or two-sided<br>*Only common tests should be described solely by name; describe more complex techniques in the Methods section.*
☒	☐	A description of all covariates tested
☐	☒	A description of any assumptions or corrections, such as tests of normality and adjustment for multiple comparisons
☐	☒	A full description of the statistical parameters including central tendency (e.g. means) or other basic estimates (e.g. regression coefficient) AND variation (e.g. standard deviation) or associated estimates of uncertainty (e.g. confidence intervals)
☐	☒	For null hypothesis testing, the test statistic (e.g. *F*, *t*, *r*) with confidence intervals, effect sizes, degrees of freedom and *P* value noted<br>*Give P values as exact values whenever suitable.*
☒	☐	For Bayesian analysis, information on the choice of priors and Markov chain Monte Carlo settings
☒	☐	For hierarchical and complex designs, identification of the appropriate level for tests and full reporting of outcomes
☐	☒	Estimates of effect sizes (e.g. Cohen's *d*, Pearson's *r*), indicating how they were calculated

Our web collection on statistics for biologists contains articles on many of the points above.

Software and code

Policy information about availability of computer code

Data collection	The phenotypes were collected using the Scan-o-matic software [Zackrisson et al. Scan-o-matic: High-Resolution Microbial Phenomics at a Massive Scale. G3. .2016 Sep 8;6(9):3003-14. doi: 10.1534/g3.116.0323442.] version 1.5.7.
Data analysis	Sequencing data were analysed using the MuLoYDH package (https://bitbucket.org/lt11/muloydh) commit cd35103 (2020/06/17) [Tattini, Lorenzo, et al. "Accurate tracking of the mutational landscape of diploid hybrid genomes." Molecular biology and evolution 36.12 (2019): 2861-2877.].<br>Other software includes:<br>R - 4.0.4 (2021-02-15)<br>R - 3.6.1 (2020/02/29)<br>NUCmer - 4.0.0 beta<br>flowCore - 1.52.1<br>LRSDAY - 1.6.0<br>fasta - 36.3.8d<br>R/qtl - 1.46-2

For manuscripts utilizing custom algorithms or software that are central to the research but not yet described in published literature, software must be made available to editors and reviewers. We strongly encourage code deposition in a community repository (e.g. GitHub). See the Nature Research guidelines for submitting code & software for further information.

Data

Policy information about availability of data

All manuscripts must include a data availability statement. This statement should provide the following information, where applicable:

- Accession codes, unique identifiers, or web links for publicly available datasets
- A list of figures that have associated raw data
- A description of any restrictions on data availability

The genome sequences generated in this study are available at Sequence Read Archive (SRA), NCBI under the accession codes: PRJNA681162 (https://www.ncbi.nlm.nih.gov/sra/PRJNA681162). The phenotype data are available within the supplementary information files. All the strains generated and stored in this work are freely available upon request.

Field-specific reporting

Please select the one below that is the best fit for your research. If you are not sure, read the appropriate sections before making your selection.

☐ Life sciences ☐ Behavioural & social sciences ☒ Ecological, evolutionary & environmental sciences

For a reference copy of the document with all sections, see nature.com/documents/nr-reporting-summary-flat.pdf

Ecological, evolutionary & environmental sciences study design

All studies must disclose on these points even when the disclosure is negative.

Study description

This study aimed at evaluating the effect of return-to-growth on the genomic and phenotypic landscape of sterile hybrids. We crossed several Saccharomyces strains to generate 20 diploid yeasts that were evolved under the return-to-growth protocol. A selection of the evolved hybrids were sequenced and their genomes characterised particularly in terms of loss-of-heterozygosity events (LOH). Finally we evaluated the fitness landscape of RTG evolved samples on a library of 128 RTG and control samples derived from a sterile intraspecific hybrid and use that resource to perform linkage analysis.

Research sample

The hybrids were derived from the following S. cerevisiae and S. paradoxus strains:
S. cerevisiae DBVPG6765
S. cerevisiae DBVPF6044
S. cerevisiae YPS128
S. cerevisiae Y12
S. cerevisiae UWOPS03-461.4
S. cerevisiae S288C
S. cerevisiae SK1
S. paradoxus N-17
S. paradoxus YPS138
S. paradoxus N-44
The research sample is representative of an interspecies population of S. cerevisiae and S. paradoxus strains.
The strains were selected according to 3 main criteria:
1) The strains were originally isolated from different ecological niches (wild and human).
2) Crossing these strains would have generated hybrids with different levels of sequence and structural divergence as well as different levels of sterility.
3) The long-read assemblies of the parental strains were available from a previous work [Yue, J. X., Li, J., Aigrain, L., Hallin, J., Persson, K., Oliver, K., ... & Liti, G. (2017). Contrasting evolutionary genome dynamics between domesticated and wild yeasts. Nature genetics, 49(6), 913-924.] and were used in the bioinformatic analysis.

Sampling strategy

The samples were available in the collection of the laboratory. The collection has been largely described in:
- Peter J, De Chiara M, et al. Genome evolution across 1,011 Saccharomyces cerevisiae isolates. Nature. 2018 Apr;556(7701):339-44.
- Yue JX, Li J, et al. Contrasting evolutionary genome dynamics between domesticated and wild yeasts. Nature genetics. 2017 Jun;49(6):913-24.

Data collection

Sequencing was performed by the Institut Curie NGS platform. For each batch, the following "instrument/flow-cell/paired-end insert-size/library-preparatation kit" were used:
A151* -> HiSeq2500 – rapid run flow cell – PE100 – Nextera XT
A314* -> HiSeq2500 – rapid run flow cell – PE150 – Nextera XT
A315* -> HiSeq2500 – rapid run flow cell – PE150 – Nextera XT
A347* -> HiSeq2500 – rapid run flow cell – PE150 – Nextera XT
A452* -> HiSeq2500 – rapid run flow cell – PE150 – Nextera XT
A505* -> HiSeq2500 – rapid run flow cell – PE150 – Nextera XT
A536* -> HiSeq2500 – rapid run flow cell – PE150 – Nextera XT
A724* -> HiSeq2500 – rapid run flow cell – PE150 – Nextera XT
A887* -> HiSeq2500 – rapid run flow cell – PE150 – Nextera XT
D287* -> NovaSeq6000 – SP flow cell – PE150 – KAPA Hyper Prep

Timing and spatial scale

No data collection on the field was performed.

Data exclusions	Low coverage (< 20 X) sequencing experiments were excluded a priori. Only one sequencing experiment was excluded since it did not meet this criterion (sequencing ID A151R2).
Reproducibility	For each hybrid, several replicates were performed (as described in Supplementary Table 6 Summary statistics of LOHs). For estimating the LOH rate with the URA3-loss assay 5 technical replicates were performed for each hybrid. Spore viability of the diploids hybrids and derived RTG samples was tested by dissecting 100 or 200 spores (Supplementary Table 3 Spore viability of the diploids hybrids and derived RTG samples). The phenotyping was performed on 8 independent technical replicates for each ScMA/ScNA sample. All attempts at replication were successful.
Randomization	Samples were not divided in groups.
Blinding	Blinding was not relevant for the study. E.g., in order to analyse the sequencing data, short-reads from evolved samples had to be mapped against the corresponding parental genomes and the results had to be compared against the corresponding control samples.

Did the study involve field work? ☐ Yes ☒ No

Reporting for specific materials, systems and methods

We require information from authors about some types of materials, experimental systems and methods used in many studies. Here, indicate whether each material, system or method listed is relevant to your study. If you are not sure if a list item applies to your research, read the appropriate section before selecting a response.

Materials & experimental systems

n/a	Involved in the study
☒	☐ Antibodies
☒	☐ Eukaryotic cell lines
☒	☐ Palaeontology and archaeology
☒	☐ Animals and other organisms
☒	☐ Human research participants
☒	☐ Clinical data
☒	☐ Dual use research of concern

Methods

n/a	Involved in the study
☒	☐ ChIP-seq
☐	☒ Flow cytometry
☒	☐ MRI-based neuroimaging

Flow Cytometry

Plots

Confirm that:

☒ The axis labels state the marker and fluorochrome used (e.g. CD4-FITC).

☐ The axis scales are clearly visible. Include numbers along axes only for bottom left plot of group (a 'group' is an analysis of identical markers).

☐ All plots are contour plots with outliers or pseudocolor plots.

☒ A numerical value for number of cells or percentage (with statistics) is provided.

Methodology

Sample preparation	Cells were first pulled out from glycerol stocks on YPD solid media and incubated overnight at 30 °C. The following day a small portion of each patch was taken with a pipette tip, transferred in 1 mL of liquid YPD and incubated overnight at 30 °C. Then, cells were washed with water, resuspended in 1 mL of cold 70% ethanol and fixed overnight at 4°C. Finally, the samples were washed twice with phosphate-buffered saline (PBS), and 100 µL of each sample were resuspended in 900µL of staining solution (15µM PI, 100µg/mL RNase A, 0.1% v/v Triton-X, in PBS) and incubated for 3hours at 37°C in the dark.
Instrument	FACS-Calibur flow cytometer
Software	The data were analyzed using FlowCore and FlowViz to divide the cell population in different bins according to the fluorescence detected with a FL2.A filter and then plotted with ggplot2 (Rversion 3.6.1) to detect the two peaks characteristics of the ploidy of the strain.
Cell population abundance	Cells were re-suspended after a overnight culture to reach a final concentration of 1000-500 cells/µL in each sample used.
Gating strategy	The distribution of fluorescence induced by staining with Propidium iodide (PI) was analysed to find the two main density peaks. These peaks correspond to the two cell populations, respectively in G1 and G2 phases and are represented as a density plot in supplementary figure 6D. The peak with the smaller values, which corresponds to the cell in G1, was used to determine the ploidy compared to a reference strain with known ploidy without gating.

☐ Tick this box to confirm that a figure exemplifying the gating strategy is provided in the Supplementary Information.
